# Relationship between waist circumference, visceral fat and metabolic syndrome in a Congolese community: further research is still to be undertaken

**DOI:** 10.11604/pamj.2013.14.20.1258

**Published:** 2013-01-14

**Authors:** Philippe Bianga Katchunga, Michel Hermans, Bertrand Akonkwa Bamuleke, Patrick Cimusa Katoto, Jeff Maotela Kabinda

**Affiliations:** 1 Département de Médecine Interne de l'hôpital Provincial Général de Référence de Bukavu (RDC); 2 Faculté de Médecine de l'Université Catholique de Bukavu (RDC); 3 Service d'endocrinologie et nutrition des Cliniques Universitaires St Luc à Bruxelles (Belgique); 4 Santé Publique-Epidémiologie, Université Catholique de Bukavu (RDC)

**Keywords:** Waist circumference, visceral fat, metabolic syndrome, Congo

## Abstract

**Introduction:**

The criteria of positivity of waist circumference to define the metabolic syndrome as currently recommended for the population of sub-Saharan Africa do not take into account specific ethnic or regional variation.

**Methods:**

The predictive value of different values of waist circumference compared with visceral fat as determined by OMRON BF510 body composition in 360 indigenous patients from Bukavu city between June 1, 2010 and May 30, 2011 was studied.

**Results:**

The prevalence was higher in women for enlarged waist circumference according to the pathological IDF or NCEP / ATP III threshold (p < 0.0001) contrasting with lower rates for pathological accumulation of visceral fat in men (p = 0.0001). The highest values for sensitivity and specificity were obtained for a threshold value of 95 cm for men (sensitivity = 72.4%, specificity = 91.1%, area under the curve (99% CI) = 0.899 (0.833 to 0.965)) and 99 cm in women (sensitivity = 75.0%, specificity = 78.3%, AUC (99% CI) = 0.844 (0.777 to 0.911)). This test also showed an independent effect on the probability of accumulation of visceral fat (Odd adjusted OR = 5.0 (99% CI: 2.1 to 11.7), p <0.0001).

**Conclusion:**

The threshold value for pathological waist circumference currently used for black African populations may overpredict abdominal fat excess in women. Further studies are needed to provide adequate cutoffs in sub-Saharan populations.

## Introduction

The prevalence of obesity and overweight is steadily increasing globally. Thus, more than one billion people may be considered as overweight, and another 400 million obese in the strict sense [1]. In the developing countries, there currently exists a true epidemiological transition for this major non-communicable disease, due to several causes, in particular as an effect of the ageing population, widespread urbanization, sedentarity, added to greater food security, along dietary changes that include enrichment in carbohydrate and saturated-lipids, frequent snacking and high-caloric density of increasingly larger meals and beverages sizes. These dietary changes associated with a decrease in energy spending related to the change in lifestyle, have resulted in the rapid emergence of obesity in the entire population [2].

In South Kivu, in the Eastern Democratic Republic of Congo, overweight/obesity affects 37.6% of the urban population versus 16.5% in rural areas. It is particularly common among urban women (46.2%) [3]. Numerous studies have demonstrated a comorbidity between obesity and other diseases, especially cardiovascular and metabolic (such as hypertension, atherogenic dyslipidemia, abnormal glucose hjomeostasis), with insulin resistance (IR) and compensatory hyperinsulinemia singled out as major common underlying etiologic factor for this cluster of cardiometabolic disorders collectively described as the metabolic syndrome (MS) phenotype [4–6].

Recent prospective studies show that cardiovascular and metabolic morbidity and mortality increase in association with the presence of a MS in both gender [7, 8]. Interestingly, all defining components of the MS are markedly improved by lifestyle intervention targeting excess caloric intake and lack of exercise. Therefore, screening for MS in the general population is of public health relevance, with the aim of delivering appropriated and targeted cardiovascular and metabolic prevention. The first proposed criteria for the diagnosis of MS required a preliminary biological confirmation of insulin resistance in patient(s) without abnormal glucose homeostasis, the latter considered as a surrogate substitute of IR due to high prevalence of reduced insulin sensitivity in patients with hyperglycemia [9, 10]. Such prerequisite hampered field screening for MS. Current definition uses a score based on five equally-weighted modifiable cardiovascular risk factors criteria easily available in routine, and all improvable by lifestyle intervention. These criteria include gender-adjusted waist circumference (WC), elevated blood pressure, and three biological parameters (hyperglycemia, hypertriglyceridemia, and gender-adjusted low HDL-cholesterol level) [11, 12].

Whereas in Europe, North-America and some Asian countries, observational studies allowed to establish a threshold value of WC associated with the MS and its determinants, equivalent data from sub-Saharan African areas are almost non-existent and incomplete in terms of their representation [13]. Moreover, ethnic and regional differences relative to central fat distribution and total white adipose tissue mass as well as to lipid and lipoproteins metabolism [14] render threshold values of WC and atherogenic dyslipidemia as currently proposed not necessarily applicable for all populations of sub-Saharan Africa.

In addition, the proposed (2009), threshold values ≥ 94 cm (men) and ≥ 80 cm (women) for sub-Saharan African populations in the consensus report of harmonization of the MS definition went nearly unnoticed [13, 15].

The present work aims to provide regional data to improve the diagnosis of MS and of its determinants in populations of sub-Saharan Africa. We determined the validity of threshold values of WC proposed by different organizations in relation to the estimated value of visceral fat, the latter determined by bioelectrical impedance, and considered as reference method for non-invasively measuring abdominal fat, whose accumulation represents the major cardiometabolic risk factor underlying the MS epidemics.

## Methods

This cross-sectional study involved patients of Black ancestry, aged ≥ 20 years and living in the province of South Kivu, who attended consultations in the Cardiology Unit of the Provincial General Hospital of Bukavu. The study period ran from June 1st, 2010 to May 30th, 2011. Subjects considered at high cardiovascular risk were included in this study, either due to the presence of ≥ 1 major classical cardiovascular risk factor (arterial hypertension, diabetes mellitus (DM), obesity, dyslipidemia) and/or of a proven cardiovascular disease (stroke, ischemic heart disease). For each patient, the medical history of arterial hypertension (AHT), DM, cardiovascular disease (stroke or ischemic heart disease) and tobacco smoking was investigated. Then, recordings of two successive measures of systolic blood pressure (SBP) and diastolic (DBP) by means of an electronic device Spengler TB-101, were performed on the right arm placed on a table, the subject being relaxed for at least five minutes in a sitting position, the cuff being fixed around the bare arm, 2 to 3 cm above elbow. The same manoeuvre was done to the left arm. The average of the two consecutive measurements was retained in this analysis. The shirtless subject was then placed standing, size and stature were measured with a meter stick, and WC was measured with a flexible tape at the end of gentle expiration, between the lower rib margins and the iliac crest.

Thereafter, weight, visceral fat, total body fat, skeletal muscle mass and body mass index were measured using the OMRON BF510 body composition monitor, which is fitted with eight applied sensors in hands and feet for accurate measurement of the electrical impedance of the whole body. An OMRON monitor equipped with such a technology, was shown to have a very good correlation with both magnetic resonance imaging (MRI) and with Dual X-Ray Absorptiometry (DEXA) for the measure of fat mass (r^2^=96%) and visceral fat (r^2^=92%), respectively [16]. At the end of the consultation, the patient was sent to the laboratory for venous blood puncture in the forearm in order to measure fasting glycemia (with fasting period ≥ 8 hours), cholesterol fractions and triglycerides. A resting electrocardiogram was also recorded. Pregnant women, subjects with edema, and bedridden were excluded from this study.

In this study, MS was defined according to the harmonized criteria of 2009 [13] based on the presence of ≥ 3 out of 5 of the following metabolic abnormalities: fasting glycemia ≥ 100 mg/dl or proven DM, blood pressure ≤ 130/85 mmHg and/or treated AHT, HDL-cholesterol 150 mg/dl in both sexes, and WC ≥ 80 cm and ≥ 94 cm in women and men respectively. The criteria proposed by the International Diabetes Federation (IDF) had as precondition the *sine qua non*presence of a WC ≤ 80 cm and ≥ 94 cm in woman and man, respectively [12]. The US criteria of the NCEP/ATP III on the other hand required a WC ≥ 88 cm and &8#8805 102 cm for men and women, respectively, without the *sine qua non*presence of enlarged waist to diagnose the MS [11]. A diagnosis of AHT was retained when systolo-diastolic blood pressure ≥> 140/90 mmHg and/or in the presence of antihypertensive therapy [17]. DM was considered in case of fasting glycemia >126 mg/dl on two occasions and/or of a history of DM [18]. In this study, the concurrence of the pair (MS + visceral fat) was selected in the presence of ≥ 2 MS criteria (blood pressure and/or biological), with the prerequisite of the presence of excess of visceral adipose tissue (visceral fat)/ (VF) as defined by impedancemetry beyond the threshold value of 10 on the manufacturer's scale, the latter covering a linear range, graded arbitrarily from 0 to 30. Similarly, the threshold values of excess body fat (BF) and reduced muscle mass (MM) were those recommended by the manufacturer.

### Statistics

The Epi Info 2000 version 3.3.2 and SPSS 17.0 softwares were used for statistical analysis, respectively. Data are presented as, means (± SD) or frequency. The Student′s t tests and chi square were used to assess the statistical significance of the observed differences. The relative contribution of different factors to the risk of accumulation of visceral or adipose tissue and that of having a pathologic abdominal measurement was assessed by multiple linear regression. The probability of an excess visceral adipose tissue according to alleged risk was modelled in a multiple logistic regression. Similarly, the predictive value of different values of waist circumference comparatively with visceral fat (gold standard) was studied, with ROC curves. The value of p ≤‘ 0.05 defined statistical significance. However, in consideration of the number of tests performed (5), results were considered conclusive with a significant difference when p ≤ 0.05/5, i.e. ≤ 0.01 (Bonferonni method).

### Ethical Considerations

The study was approved by the Ethics Committee of the School of Medicine of Bukavu Catholic University. Informed consent was obtained from all patients included in the study.

## Results

### General characteristics of the population studied

General characteristics of participants are shown in Table 1. During the study period, 360 patients consulted for assessment of their cardiovascular risk due to the presence of at least one major classical cardiovascular risk factor and/or due to established cardiovascular disease. The average age was 52.4± 13.4 years for men and 49.1 ± 11.8 years for women (p = 0.01). In this group, BMI (kg/m^2^) was higher in women than in men (women vs. men: 29.2 ± 5.8 vs. 25.9 ± 4.0, p10/30) were older and had a BMI, a W.C and an adipose tissue mass, higher than patients with visceral fat < 10/30 (p <0.0001), alongside a lower skeletal muscle mass (p = 0.0008).

 SBP and DBP values were higher in patients with visceral fat >10/30 (p <0.01), a subgroup in which total cholesterol (mg/dl) (219.4 ± 54.8 vs. 198.3 ± 58.5, p = 0.0005), low-density lipoprotein cholesterol (LDL-C; mg / dl) (147.8 ± 51.4 vs. 128.1 ± 47.0, p=0.0003) and triglycerides (mg/dl) (134.1 ± 78.0 vs. 110.7 ± 61.3, p=0.001) were also higher.

**Table 1:** General characteristics of the study population

	Whole group	Men	Women	p	Visceral Fat ≥10/30	Visceral Fat <10/30	p
**Number (%)**	360 (100)	143 (39,7)	217 (60,3)		175 (48,6)	185 (51,4)	
**Averages (±SD)**							
Age, years	50,4±12,5	52,4±13,4	49,1±11,8	0,01	54,4±9,6	46,7±13,8	<0,0001
BMI, Kg/m^2^	27,9±5,4	25,9±4,0	29,2±5,8	<0,0001	30,8±4,8	25,1±4,3	<0,0001
WC, cm	94,9±13,4	93,9±12,5	95,6±13,9	0,24	102,5± 11,2	87,7±11,1	<0,0001
B.F,%	36,1±11,8	25,8±7,6	42,9±8,7	<0,0001	39,3±10,2	33,0±12,3	<0,0001
V.F,%	9,7±4,1	10,8±4,8	9,0±3,3	0,0001	–	–	–
M.M,%	27,7±5,9	33,0±4,9	24,3±3,5	<0,0001	26,7±5,0	28,8±6,5	0,0008
T.C, mg/dl	208,5±57,7	208,2±49,6	208,8±62,5	0,92	219,4±54,8	198,3±58,5	0,0005
HDL-C, mg/dl	46,5±16,4	44,4±12,1	47,9±18,6	0,0490	45,5± 13,9	47,4±18,5	0,26
LDL-C, mg/dl	137,4±50,1	139,6±47,2	136,1±51,8	0,53	147,8±51,4	128,1±47,0	0,0003
TG, mg/dl	122,0±70,8	123,3±84,8	121,2±60,0	0,77	134,1±78,0	110,7±61,3	0,001
Glycemia, mg/dl	104,3±43,2	104,2±41,1	104,4±44,9	0,97	105,0±44,8	103,6±41,8	0,79
SBP, mmHg	146,0±24,5	148,8±23,1	144,1±25,3	0,07	151,6±23,6	140,7±24,3	<0,0001
DBP, mmHg	87,1±15,4	90,1±15,5	85,1±15,0	0,002	90,0±14,6	84,2±15,6	0,0003
**Frequency (%)**							
Advanced age	25,0	46,2	11,1	<0,0001	33,1	17,3	0,0005
AHT	75,6	78,3	73,7	0,32	83,4	68,1	0,0007
DM	31,8	28,9	33,9	0,37	33,1	30,6	0,65
Obesity	34,6	19,7	44,4	<0,0001	52,9	17,4	<0,0001

WC= Waist circumference, B.F= Body fat, V.F= Visceral fat, MM= Muscle mass, T.C= Total Cholesterol, HDL-C= High density lipoprotein cholesterol, LDL-C= low density lipoprotein cholesterol, TG= triglycerides, SBP= Systolic blood pressure, DBP= Diastolic blood pressure, AHT= Arterial hypertension, DM= Diabetes mellitus

### Cardiovascular risk factors

**Figure 1:**
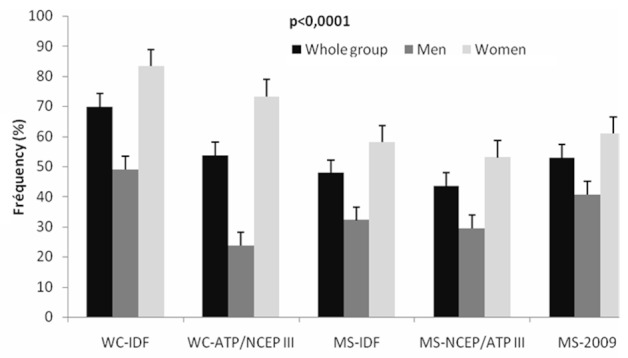
Frequency (%) of pathological waist circumference and metabolic syndrome in the whole group, in men and women respectively, according to the IDF, NCEP/ATP III and harmonized in 2009 criteria

**Figure 2:**
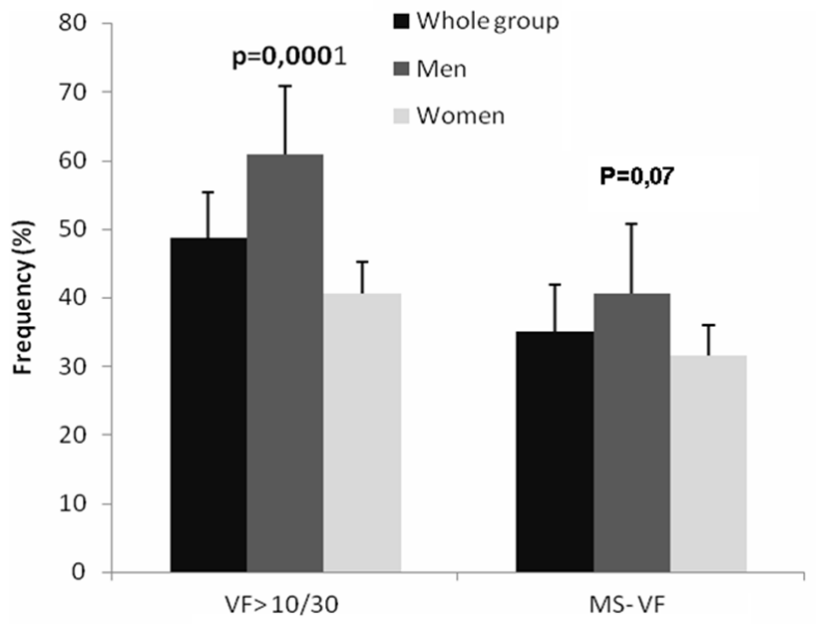
Frequency of the perivisceral fat accumulation measured by electrical impedance and the metabolic syndrome according to the perivisceral fat accumulation in the whole group, respectively, in men and women

Table 1 and Figure 1 and Figure 2 reproduce the prevalence of cardiovascular risk factors. Smoking (3.9% in the whole group) was more prevalent in men (8.5% vs. 0.5% in women, p = 0.0002). In the entire group, 75.6% of patients were hypertensive and 31.8% were diabetic. Obesity, defined as a BMI >30.0 kg/m^2^; was present in 34.6%, being more common in females than in males (44.4% vs. 19.7%, p = 10/30 (48.6%) was more predominant in males (60.8%) than in female (40.6%) (p = 0.0001). There was no statistically significant difference as to the frequency of MS according to the accumulation of visceral fat (35.1%) among men (40.6%) and women (31.5%) (p = 0.07).

### Predictive value of waist circumference

ROC curve construction (Table 2 and Table 3) shows that for the test of a WC (cm) in the visceral accumulation of adipose tissue estimated by bioelectrical impedance (gold standard), the area under the curve (99% CI) is respectively from 0.899 (0.833 to 0.965) and 0.844 (0.777 to 0.911) in men and in women. A threshold value (criteria of positivity) of 80 cm had a sensitivity of 100.0% in women and from 93.0% in men. By cons, a threshold value of 105 cm, respectively, had a specificity of 100.0% and 78.7% in men and women.

**Table 2:** Predictive value of waist circumference in women patients

	Area under the curve (99% CI)	Sensitivity (%)	Specificity (%)	100%-specificity	Youden index
W.C> 80,5 cm	0,844 (0,777–0,911)	100,0	29,5	70,5	0,295
W.C> 88,5 cm		94,3	48,1	51,9	0,424
W.C> 90,5 cm		93,2	54,3	45,7	0,475
W.C> 94,5 cm		84,1	64,3	35,7	0,484
W.C> 98,5 cm		75,0	78,3	21,7	0,533
W.C> 100,5 cm		64,8	84,5	15,5	0,493
W.C> 105,5 cm		47,7	93,0	7,0	0,407

WC= Waist circumference

**Table 3:** Predictive value of waist circumference in men patients

	Area under the curve(99% CI)	Sensitivity (%)	Specificity (%)	100%-specificity	Youden index
W.C> 80,0 cm	0,899 (0,833–0,965)	97,7	32,1	67,9	0,298
W.C> 88,5 cm		90,8	67,9	32,1	0,587
W.C> 90,5 cm		83,9	76,8	23,2	0,607
W.C> 94,5 cm		72,4	91,1	8,9	0,635
W.C> 98,5 cm		54,0	96,4	3,6	0,504
W.C> 100,5 cm		39,1	98,2	1,8	0,373
W.C> 105,5 cm		27,6	100,0	0,0	0,276

WC= Waist circumference

However, the highest values for the sensitivity and the specificity were obtained with a threshold value of 95 cm (rounded from 94.5 cm) in male patients (sensitivity = 72.4%, specificity = 91.1%, Youden index = 0.63), and 99 cm in female patients (sensitivity = 75.0%, specificity = 78.3%, Youden index = 0.53).

### Determinants of visceral fat and of waist circumference

In the multiple linear regression, 76.1% of variability in visceral fat was explained by age (regression coefficient per year, 0.077, p <0.0001), BMI (per kg/m^2^ 0.467, p <0.0001) and WC (per cm, 0.094, p = < 0.0001). For WC, 80.0% of variability was explained by visceral fat (regression coefficient by 0–30 graduation, 0.873 cm, p < 0.0001) and BMI (kg/m^2^ , 38 cm <0.0001).

In the multiple logistic regression (Table 4), the criterion of positivity in patients 95 cm and 99 cm in patients showed an independent effect on the probability of visceral accumulation of adipose tissue (Odd adjusted OR = 5.0 (CI 99%: 2.1 to 11.7), p <0.0001) after adjustment for advancing age, male gender, obesity, higher body fat and higher muscle mass.

**Table 4:** Multivariable-Adjusted Odds Ratios for perivisceral accumulation of adipose tissue

Independent variable	VF>10/30 / VF<10/30 prevalence rates (%)	Unadjusted OR (99% CI)	p	Adjusted OR (99% CI)	p
Advanced age	64,4/ 43,3	2,3 (1,44– 3,88)	0,0006	8,7 (2,1-35,6)	0,0001
Obesity	74,2/ 35,0	5,3 (3,28- 8,64)	0,0000	3,7 (1,4-9,8)	0,0004
Hign BF	59,9/ 6,6	21,1 (8,29- 54,06)	0,0000	43,9 (6,8-283,1)	<0,0001
W.C> 95 (M)/ 99 (F) cm	79,6/ 23,2	12,9 (7,79-21,40)	<0,0000	5,0 (2,1-11,7)	<0,0001
Male	60,8/ 40,6	2,2774 (1,4793 3,5062)	0,0002	5,6 (2,1-11,7)	<0,0001
High MM	8,1/ 53,1	0,07 (0,02 -0,25)	0,0000	0,8 (0,09–7,63)	0,85

WC= Waist circumference, B.F= Body fat, V.F= Visceral fat, MM= Muscle mass

## Discussion

This cross-sectional study documented the relationship between waist circumference and visceral fat measured by electrical impedance in a group of 360 Black sub-Saharan African patients from South Kivu with high cardiovascular risk due to the presence of at least one major cardiovascular risk factor and/or cardiovascular disease.

Several relevant observations were made, among which the expected high correlation between waist circumference and visceral fat. This was reported by many authors, and measuring waist circumference is considered a valid non-expensive and non-invasive surrogate to assess the combination of visceral and peri-visceral adipose tissue [14, 15, 19–21]. However, in the subgroup of women from this series, in whom the proportion with pathological waist circumference according to IDF or NCEP/ATP III was very high, the frequency of visceral fat accumulation as determined by electrical impedance was rather low. This corroborates the observations of Wildman et al. [22], who noted that waist circumference was similar in both insulin-sensitive and insulin-resistant obese patients. In addition, Meigs et al. [23] noted that 75% of their obese patients without MS nevertheless had an elevated waist circumference. Besides, a study conducted in South Africa showed that 38% of women with proven obesity were found to be normally sensitive to insulin, and otherwise had an unremarquable cardiometabolic profile, corresponding to the *Metabolically Healthy but Obese*phenotype [24].

It comes out from this series that a significant proportion of Black women with pathological abdominal perimeter would not *a priori* present a phenotype associated with insulin resistance nor with accumulation of visceral adipose tissue. Among confounding factors related to measurement of abdominal perimeter, one should not overlook that a substantial part of the circumference measured incorporates the contribution of a corona of subcutaneous adipose tissue [14], physiologically well-developed in the female gender, and moreover that the measure of the abdominal perimeter could be artificially altered as a result of decreased parietal muscle tonicity associated with widespread multiparity observed in sub-Saharan areas. Another relevant observation is the demonstration of rather satisfactory average routine lipid values in the whole group, particularly as regards triglycerides. This was previously reported in Black populations, both from indigenous populations and from ancient or recent ethnic migrants subgroups, such as African-Americans [25–27].

Studying the predictive value of abdominal measurement comparatively to visceral accumulation of fat by impedancemetry showed that only a threshold value of 80 cm had a very high sensitivity in both sexes, but with a very low specificity [28].

This is consistent with the observations of other authors, who emphasized the importance of taking into account, for establishing a pathological waist circumference, the marked regional and ethnic disparities which are highly prevalent in such an immense territorial and ethno-cultural cradle as that of sub-Saharan Africa.

The current criteria defining the MS arose from a laborious consensus among key working groups. However, the paucity of recent reliable data representative of many parts of Africa prompted the scientific community to strangely recommend until recently the use, by default, of European Caucasian threshold values for the African environment. In this context, one should mention the work of Longo-Mbenza et al. [29], in the DR Congo, who noted that the criteria of the IDF with a threshold value of 94 cm in both sexes appeared more appropriate for that African area. In the same way, a South-African study showed that threshold values of 92 cm in females and 86 cm in males, better predicted the presence of two other parameters of the metabolic syndrome [30].

The major limitation of this work is its transversal nature, which does not allow to study causal links between waist circumference, visceral fat and the occurrence of new-onset cardiovascular events and cardiometabolic diseases. Similarly, the reference method of planimetry CT or DEXA to measure visceral fat was not used for the obvious reason of non-availability. This is why despite a highly statistically significant correlation between body composition determined by Tetrapolar bioelectrical impedance OMRON BF 500 and whole body magnetic resonance imaging (MRI) and dual energy X-ray absorptiometry (DEXA) in healthy normal weight, overweight and obese adults, bio-impedencemetry merely provides with a qualitative index of peri-visceral fat [16]. In addition, the exclusive study of subjects at high cardiovascular risk may be biased by certain errors due to the change of body composition, including drug-induced changes as a result of diuretics and antidiabetic therapies. According to the latter, generalizing these results to the whole population is discussable.

In addition, the huge cultural and ethnic diversity of the people of the DRC does not authorize any general extrapolation of the present results, gathered in one area of Eastern Congo to the entire nation. In spite of these limitations, the present study provides relevant and practical observations, which may help improve and refine the definition of MS in Congolese areas.

## Conclusion

This study, comparing threshold value of waist circumference and measurement of visceral fat by electrical impedance, demonstrates that current metabolic syndrome criteria may substantially overpredict excess abdominal obesity, especially among women, in Congolese areas. Further studies are needed to provide adequate cutoffs in sub-Saharan populations.
